# Functional morphology of the leg musculature in the marine seal louse: adaptations for high-performance attachment to diving hosts

**DOI:** 10.1038/s41598-025-32804-2

**Published:** 2025-12-23

**Authors:** Anika Preuss, Thomas van de Kamp, Elias Hamann, Marcus Zuber, Lina Ornowski, Stanislav N. Gorb

**Affiliations:** 1 Department of Functional Morphology and Biomechanics, Zoological Institute, Kiel University, Am Botanischen Garten 1–9, 24118 Kiel, Germany; 2 Institute for Photon Science and Synchrotron Radiation (IPS), Karlsruhe Institute of Technology (KIT), Hermann-Von-Helmholtz-Platz 1, 76344 Eggenstein-Leopoldshafen, Germany; 3 Laboratory for Applications of Synchrotron Radiation (LAS), Karlsruhe Institute of Technology (KIT), Kaiserstr.12, 76131 Karlsruhe, Germany

**Keywords:** Parasitism, Seal louse, Human head louse, Marine mammals, Biomechanics, Extremities, Skeleton-muscle organization, Ecology, Ecology, Zoology

## Abstract

The seal louse (*Echinophthirius horridus*) is a remarkable example of evolutionary adaptation, thriving as an obligate ectoparasite on deep-diving marine mammals under extreme environmental conditions, including high hydrostatic pressure, extreme drag force, salinity, and fluctuating temperatures. To investigate the anatomical and functional specializations enabling this lifestyle, we compared the leg morphology and musculature of *E. horridus* with its terrestrial relative, the human head louse (*Pediculus humanus capitis*), using synchrotron-based 3D microtomography and confocal laser scanning microscopy. Our findings reveal that the seal louse has developed a highly compact and robust leg structure with a fused tibiotarsus, an additional set of leg muscles, and a shortened claw tendon—an unprecedented adaptation among insects. These features allow for greater force transmission and reduced metabolic cost during sustained attachment. Behavioral assays further show that *E. horridus* can only move effectively on hair-like substrates, underscoring its complete reliance on host fur. These findings suggest a highly specialized muscular control system enabling strong, reliable, and reversible attachment in a challenging aquatic environment.

**Supplementary Information:**

The online version contains supplementary material available at 10.1038/s41598-025-32804-2.

## Introduction

Secure attachment to various surfaces is crucial for many organisms for such functions as settlement, copulation, locomotion, parasitism or phoresy, sticking body parts to one another or for maintenance of position^1^. The morphology of attachment devices is typically heavily influenced by the species’ biology and the specific biological function of these devices^2–4^. Eight different attachment mechanisms have been identified so far: (i) suction, (ii) hooks, (iii) dry adhesion, (iv) clamp, (v) wet adhesion (capillarity, cement/glue), (vi) lock or snap, (vii) friction, and (viii) spacer or expansion anchor^1^. At first glance, many of these attachment mechanisms appear to function passively, for example by hooking a claw into the substrate or by glue as seen in many sessile animals including sponges, bivalves, insect pupae or phoretic mites^5–7^. However, many of these attachment mechanisms can only function, if they are actively controlled by muscles, in addition to passive mechanisms such as claw interlocking. A famous example are geckos, which are known to use microscopic setae to employ attractive van der Waals forces of the superhydrophobic adhesive toe pads for attachment to various substrates^8–16^. Although this might seem like a passive process, the attachment system is actually organized in a hierarchical manner, incorporating intricate musculo-tendinous, vascular, and sensory components that are essential for establishing attachment, adjusting the strength of the attachment, and eventually facilitating detachment^6,17,18^. Other examples include aquatic animals, such as leeches, octopuses, and diving beetles that use suction cups for secure underwater attachment, which is either produced actively by muscular action or by recoiling elastic elements causing passive suction, both expanding the volume under the suction cup and therefore generating a negative pressure that secures attachment underwater^6,19–23^.

In insects and spiders, the claw retractor muscle can induce (1) normal forces on the adhesive pads, producing local deformations of the cuticle and increasing the contact area with the substrate, and (2) detachment upon muscle relaxation^6,24–30^. Contraction of the *retractor unguis* muscle also pulls on the unguitractor plate, which in turn transmits and distributes forces to the claw. Studies in other insects show that the unguitractor apparatus can provide frictional stabilization and delayed return of the claw when external substrate resistance is present, thereby reducing the muscular effort required to maintain a gripping posture^31^. However, this behavior does not represent a fully passive self-locking mechanism: the claw cannot remain bent without baseline muscle tension, and detachment occurs rapidly when muscular activity ceases. Thus, the unguitractor plate modulates and supports, but does not replace, active muscular control during attachment^31^.

Upon examining the attachment forces, by which animals adhere to various surfaces, it becomes evident that the habitat and lifestyle significantly influence the magnitude of these forces: while non-parasitic animals, such as hoverflies (*Sphaerophoria scripta* and *Episyrphus balteatus;* Syrphidae, Diptera), usually show maximum attachment forces of about 25–30 times their own body weight (safety factor)^32^, the attachment forces of parasites usually by far exceed these values. For example, the bee ectoparasite *Braula coeca* (Braulidae, Diptera) and the avian ectoparasite *Crataerina pallida* (Hippoboscidae, Diptera) generate safety factors of about 1000–3000 by using comb-like structures and tridentate claws to interlock with their host hairs or feather barbs^33,34^. However, these values are exceeded by far by the seal louse, a semi-aquatic parasitic insect living in the fur of harbor seals, reaching safety factors up to 18,000 even in the marine environment^35^. Echinophthiriidae (Phthiraptera: Anoplura) are obligate ectoparasites of pinnipeds living in the fur of their hosts and feeding on their blood^36,37^. As their hosts returned from land to sea during the Miocene, they had to adapt to a challenging new marine environment with fluctuating temperatures, high salinity, extreme hydrostatic pressure and hypoxia^38–40^. The seal louse, *E. horridus*, parasitizes harbor seals (*Phoca vitulina*) and grey seals (*Halichoerus grypus*)^41–43^. During diving activities, these pinnipeds are capable of descending to depths between 450 and 631 m, remaining submerged for durations of up to 35 min^44–49^, and encountering water temperatures as low as 0 °C^50,51^. Although these values refer to the surrounding water, the temperatures experienced by their ectoparasites likely approach, but do not necessarily equal, these conditions due to thermal buffering by the host. Furthermore, during their routine haul-outs on land, they are exposed to temperatures reaching up to 28.6 °C^51–53^. Consequently, in addition to confronting these substantial temperature fluctuations and a hydrostatic pressure of approximately 60 kg*cm^-2^ (5883.96 kPa) at a depth of 600 m^54^, the seal lice residing on the seals’ surface must also maintain a secure attachment to the seals’ fur, even as their hosts swim at velocities of 18 km/h^55^. These values represent the swimming speed of the seal and not the precise flow velocities within the fur where the lice reside. Seal lice are embedded in the turbulent boundary layer that forms close to the skin and around the flattened seal hairs; in this region, flow velocities are attenuated compared to the external free-stream flow, yet characterized by high shear and local turbulence. Consequently, the lice do not experience the full ambient velocity, but they are nevertheless subjected to substantial and rapidly fluctuating hydrodynamic forces^56^. They counteract these forces through a sophisticated attachment mechanism based on a modified snap-hook system, in which a strongly sclerotized claw clamps a seal hair securely between itself and a thumb-like counterpart^35^. This system is supported by two pads on the inside of the tibiotarsus complex, the tibial pad and the euplantula. These are made of soft, probably resilin-containing material and thus presumably capable of increasing friction on the seal hair, when the claw is closed around the hair. This mechanism enables them to achieve extremely high safety factors of up to 18,000^35^.

However, it remains unclear how this entire mechanism is controlled. Is it merely a passive clasping or an active holding on to the hair? Is secure attachment maintained mainly by continuous muscle activity, or do structural elements like the unguitractor plate also contribute to grip stability? How does this mechanism function in comparison to terrestrial, closely related Anoplura? Is it a special adaptation to the marine way of life? To address these questions, we compared the leg musculature of the seal louse, *E. horridus*, with that of the terrestrial human head louse, *Pediculus humanus capitis*, which represents a closely related anopluran species and therefore provides an appropriate terrestrial reference for distinguishing marine-specific adaptations. Modern 3D reconstructions based on synchrotron X-ray microtomography were used to visualize and analyze the musculature in both species. Additionally, locomotion of seal lice on different substrates was examined using video recordings of live individuals, and general body and leg morphology was compared using confocal laser scanning microscopy. This study thereby elucidates the mechanisms that enable robust, reliable, and reversible attachment to complex structures of the host in the deep ocean.

## Materials and methods

### Animals

During necropsies of harbor seals (*P. vitulina*) and grey seals (*H. grypus*) found dead or dying along the Baltic Sea coast in Schleswig–Holstein from April to November 2022, seal lice (*E. horridus*; Anoplura; Insecta) were collected. The specimens analyzed in this study were sourced from seals examined as part of Schleswig–Holstein’s stranding network monitoring programs to evaluate the health condition of the seals^57–59^. Ethical review and approval were not required for this study, as all host animals were either found dead, died naturally, or were euthanized on welfare grounds, with none being killed specifically for this research. The authors were not involved in the euthanasia of the hosts, which was carried out by certified seal rangers for reasons unrelated to this study. All regulations regarding animal use were strictly followed. In 2023, human head lice were collected in Göttingen, and preserved in 70% ethanol.

### Confocal laser scanning microscopy (CLSM)

For CLSM analysis, seal lice (n = 2) and human head lice (n = 2) were placed in glycerine (≥ 99.5%) and covered with a high-precision cover slip (thickness = 0.170 mm ± 0.005 mm, refractive index = 1.52550 ± 0.00015, Carl Zeiss Microscopy GmbH, Jena, Germany) before scanning. The samples’ natural fluorescence was assessed using a CLSM Zeiss LSM 700 equipped with an upright Zeiss Axio Imager microscope (Carl Zeiss Microscopy GmbH, Jena, Germany). Four solid-state lasers with wavelengths of 405 nm, 488 nm, 555 nm, and 639 nm, along with corresponding emission filters (BP420–480, LP490, LP560, LP640 nm), were utilized. The 405 nm excitation and 420–480 nm emission filter highlighted less sclerotized cuticle, potentially rich in resilin^60^. Regions with higher sclerotization were identified using 488 nm and 555 nm laser excitations with filters that allowed emission light with wavelengths above 490 nm and above 560 nm, respectively. The 639 nm laser excitation with a 640 nm long-path emission filter captured extended autofluorescence. Projections were processed using ZEN 2008 software (www.zeiss.de/mikroskopie) and Adobe Photoshop CS6 (Adobe Photoshop CS, San José, USA) for qualitative, not quantitative, analysis of cuticle composition^60–64^. The distinct colors seen in the autofluorescence images are linked to specific material properties^60^ as follows. Reddish autofluorescence indicates highly sclerotized cuticle, with more intense red hues suggesting greater sclerotization. Greenish autofluorescence is associated with relatively resilient cuticle with a high chitin content, while bluish autofluorescence indicates softer, less-sclerotized regions, often containing resilin.

### Synchrotron x-ray microtomography and 3D reconstruction

Specimens of *E. horridus* and *P. humanus capitis* (n = 1) were examined in 70% ethanol at the IMAGE beamline^65^ within the Imaging Cluster at KIT Light Source. The beam generated by the superconducting wiggler was filtered using 2 mm pyrolytic graphite and monochromatized at 18 keV with a Double Multilayer Monochromator (DMM). We utilized a rapid indirect detector system that included a scintillator, visible light optics, a white beam microscope (Optique Peter, Lentilly, France)^66^, and a 12-bit pco.dimax high-speed camera (Excelitas PCO GmbH, Kelheim, Germany) featuring 2016 × 2016 pixels with a physical size of 11 µm. A 10 × magnification provided an effective pixel size of 1.22 µm. For each scan, 200 dark field images, 200 flat field images, and 3000 equiangularly spaced radiographic projections over a 180° range were captured at a frame rate of 50 fps. The control system Concert^67^ was used for automated data collection. Data processing, including dark and flat field correction and phase retrieval, was conducted using the UFO framework^68^. The final tomograms were reconstructed with tofu software^69^, resulting in phase and absorption contrast data sets. These were combined and converted into 8-bit volumes. Data segmentation was performed using Amira 6.2.0 (Thermo Fisher Scientific, Waltham, US) and visualized with Blender 3.4 (Blender Foundation, Amsterdam, Netherlands).

### Video recordings on different substrates

An Olympus SZX10 stereomicroscope (Olympus, Tokyo, Japan) including a SDFPLAPo2XPFC 14–230 × magnification objective lens in brightfield mode was used to take videos of seal lice (n = 5, 25 fps) moving freely on polishing paper of different roughness (0.3, 1 and 12 µm; FibrMet® Abrasive Discs, Buehler, Lake Bluff, USA), on seal hair, and on human hair.

## Results

### General morphology of the body of *E. horridus *and *P. humanus capitis*

**Fig. 1:**
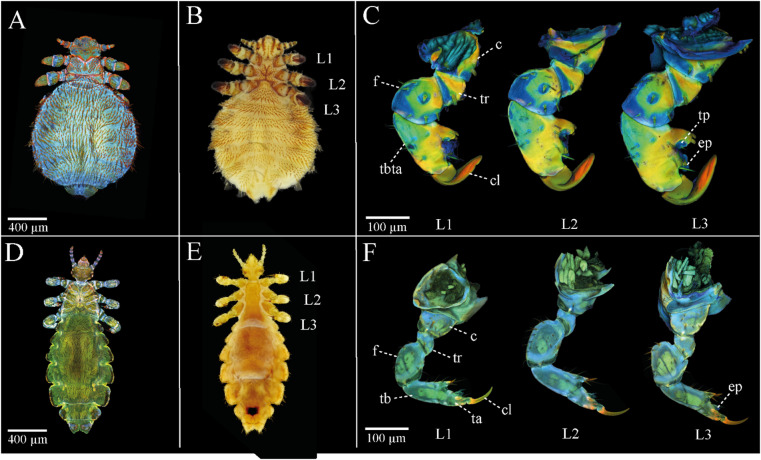
General habitus and leg morphology (legs 1–3) of *E. horridus* and *P. humanus capitis*. (**A**,** B**) General habitus of *E. horridus* from dorsal view (**A**, CLSM maximum intensity projection) and from ventral view (**B**, light microscopical image); (**C**) CLSM maximum intensity projections of the legs (L1-3) of *E. horridus* from lateral view; (**D**,** E**) General habitus of *P. humanus capitis* from dorsal view (**D**, CLSM maximum intensity projection) and from ventral view (**E**, light microscopy image); **F**) CLSM maximum intensity projections of the legs (L1-3) of *P. humanus capitis* from lateral view. The distinct colors seen in the autofluorescence images are linked to specific material properties ^60^ as follows. Reddish autofluorescence indicates highly sclerotized cuticle, with more intense red hues suggesting greater sclerotization. Greenish autofluorescence is associated with relatively resilient cuticle with a high chitin content, while bluish autofluorescence indicates softer, less-sclerotized regions, often containing resilin. Abbreviations: c (coxa), cl (claw), ep (euplantula), f (femur), ta (tarsus), tb (tibia), tbta (tibiotarsus complex), tp (tibial pad), tr (trochanter).

The body of Anoplura is generally divided into three sections, as typical in insects: head, thorax, and abdomen, with three pairs of legs originating from the thorax (Fig. 1). The bodies of both lice species exhibit a notable blue autofluorescence, which suggests a low degree of sclerotization and a flexible cuticle (Fig. 1A, B, D, E).

The legs of the seal louse and the human head louse vary in length with the anterior legs being more slender and shorter than the more posterior ones. Within each species, all legs share the same number of segments: in the seal louse, each leg consists of four segments plus a claw (coxa, trochanter, femur, and a fused tibiotarsus complex), whereas in the human head louse each leg comprises five segments plus a claw (coxa, trochanter, femur, tibia, tarsus) (Fig. 1). The seal louse’s leg cuticle is consistently more sclerotized in the proximal region of each leg segment near the joints. In contrast, the distal side of each segment, which overlaps with the proximal side of the next segment, is less sclerotized^35^ (Fig. 1C). Furthermore, the tibiotarsus complex is highly sclerotized, except for the euplantula and the tibial pad showing prominent blue autofluorescence and therefore probably soft cuticle material (Fig. 1C). In the human head louse, the reddish autofluorescence is largely confined to the tarsus and claw region, while the other leg segments are mainly dominated by greenish to bluish autofluorescence indicating less sclerotized regions in comparison to the more sclerotized distal segments (Fig. 1F). We could not detect any structure resembling the described tibial pad in *E. horridus,* but an euplantula, which is also dominated by bluish autofluorescence and therefore merely distinguishable from the surrounding cuticle of the tibia (Fig. 1F). To improve clarity, a magnified view of this region is provided in Supplementary Figure S1. In *E. horridus*, the claw is generally more curved and robust compared to that of *P. humanus capitis*. Additionally, the thumb-like counterpart of the claw in the seal louse is significantly more developed, featuring short, stocky stopper setae, in contrast to the slender thumb-like counterpart with elongated, more fragile setae observed in the human head louse (Fig. 1C & F).

### Locomotion on various substrates

Upon examining the video recordings across various substrates, it was observed that seal lice exhibited limited mobility on flat surfaces with differing degrees of roughness. The claws failed to secure a grip on the flat substrate, irrespective of its texture, and the abdomen remained in contact with the surface (Supplementary Video S2). Conversely, on seal hair, the lice demonstrated significant mobility, consistently seeking a foothold with their anterior pair of legs prior to advancing the remaining pairs, showing a tripod-gait pattern (Supplementary Video S3). Notably, the lice were equally adept at walking on strongly curved cylindrical substrates, such as human hair, even employing unilateral movement, wherein only three legs maintained contact with the substrate. The legs on the opposite side moved synchronously in the air without substrate contact, yet this unilateral contact sufficed to facilitate secure and efficient movement along the hair (Supplementary Video S4).

### Comparison of leg musculature of *E. horridus* and *P. humanus capitis*

**Fig. 2:**
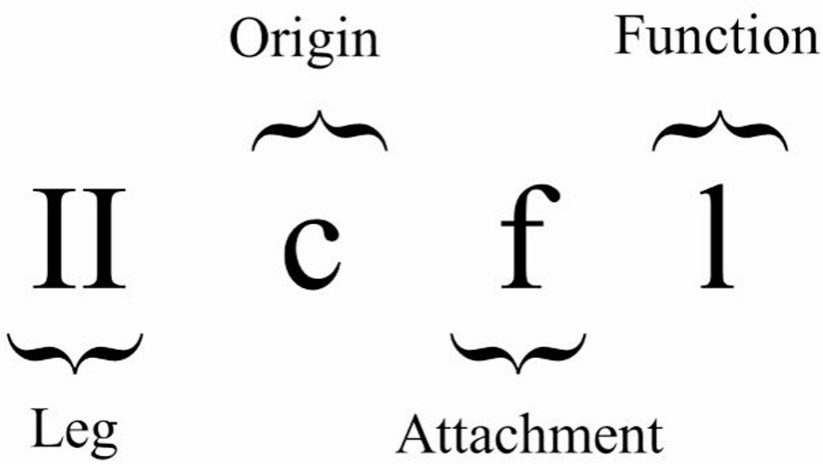
The leg muscles of *E. horridus* and *P. humanus capitis* are named according to Gray et al. ^70^. The legs are enumerated using Roman numerals, commencing from the anterior. Subsequently, the origin and insertion points of the muscle are specified, followed by the identification of its function. The corresponding abbreviations are delineated in Table 1. In this particular case, the muscle under consideration is located in the second leg (II), originates from the coxa (c), attaches to the femur (f), and serves as a levator (l).

Due to the bilateral symmetry of the body, the descriptions and analyses presented here are confined to the right side of the body of an adult female seal louse and a human head louse. The muscle terminology utilized is based on Gray et al.^70^ and encompasses the following components: (i) leg segment, (ii) muscle origin, (iii) muscle insertion, and (iv) muscle function (Fig. 2).

Muscles can be classified according to their functions into flexors (fl), which facilitate the bending of the leg; extensors (e), that assist in straightening the leg; depressors (d), which pull the leg downward; levators (l), that elevate the leg upward; protractors (p), which move the leg forward; and retractors (r), which draw the leg backward (Table 1).

**Table 1:** List of abbreviations for the names of the leg muscles of *E. horridus* and *P. humanus capitis* according to Gray et al. ^70^.

Leg	Origin and attachment		Function	
I	c	Coxa	fl	Flexor
II	tr	Trochanter	e	Extensor
III	f	Femur	d	Depressor
	tb	Tibia	l	Levator
	ta	Tarsus	p	Protractor
	tbta	Tibiotarsus	r	Retractor
	cl	Claw		
	fu	Furca		
	ut	Unguitractor plate		
	clt	Claw tendon		
	ct	Coxal tendon		
	vct	Ventral coxal tendon		
	dct	Dorsal coxal tendon		

**Fig. 3:**
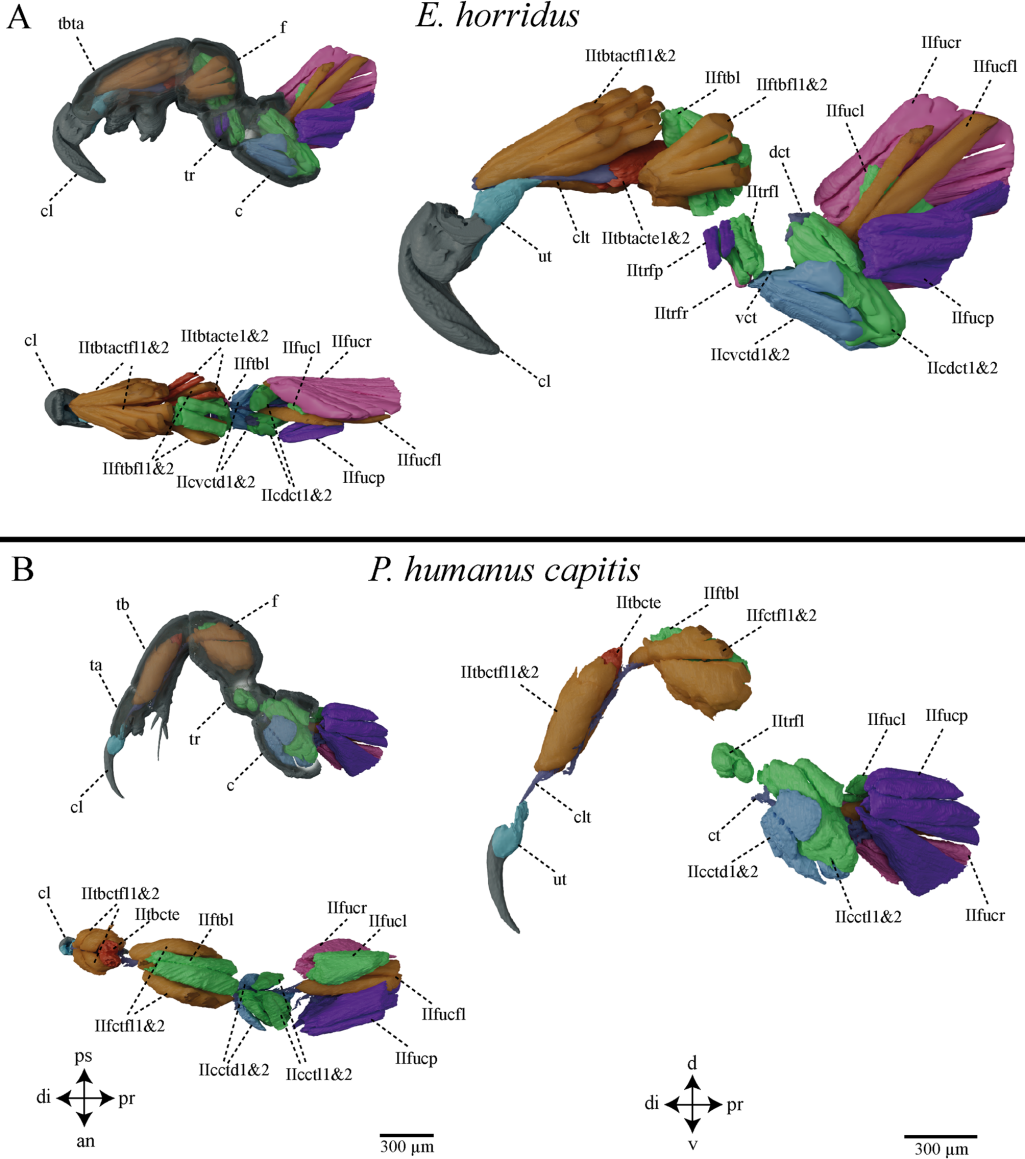
Extrinsic and intrinsic musculature of the second leg of *E. horridus* (**A**) and *P. humanus capitis* (**B**). **A**) Musculature of the second leg of *E. horridus* with transparent cuticle from lateral view, without cuticle from lateral view and without cuticle from dorsal view. **B**) Musculature of the second leg of *P. humanus capitis* with transparent cuticle from lateral view, without cuticle from lateral view and without cuticle from dorsal view. Abbreviations: see Table 1 & Supplementary Table S7; anterior (an), dorsal (d), distal (di), proximal (pr), posterior (ps), ventral (v).

Within each species, no differences in the musculature were observed among the individual legs; all legs exhibit a uniform structural organization (Fig. 3; Supplementary Figures S5 & S6). A complete overview of the musculature, including the anatomical notation and all muscle abbreviations, is provided in Supplementary Table S7. In the main text, we focus on the muscles that differ between the two species. These interspecific differences include the following:

I/II/IIItbtacte1&2 – The second extensor muscle is only present in the seal louse.

I/II/IIItrfp – This protractor is only developed in the seal louse.

I/II/IIItrfr – This retractor is only developed in the seal louse.

tbta – The tibia and tarsus are fused in the seal louse to a tibiotarsus complex.

clt – The claw tendon of the seal louse ends in the middle of the tibiotarsus complex and not at the beginning of the femur as observed in the human head louse.

vct – This tendon can only be found in the seal louse.

dct – This tendon can only be found in the seal louse.

## Discussion

### Comparison of body and leg morphology of *E. horridus* and *P. humanus capitis*

In general, the body of the seal louse is much more compact and rounded than that of the terrestrial head louse (Fig. 1). A direct comparison of body volumes and surface areas shows that the seal louse possesses approximately twice the body volume and surface area of the head louse despite having a similar overall body length^71^. This trend is consistent with recent comparative analyses in echinophthiriid lice, which demonstrate that species associated with deeper-diving hosts tend to exhibit increasingly rounded body shapes^72^. A more compact, globular morphology likely provides improved mechanical stability in a highly dynamic underwater environment by distributing external mechanical loads more uniformly across the body surface. Such stability may be advantageous not only under elevated hydrostatic pressures but also within the turbulent boundary layer along the host’s body during swimming^72^.

Importantly, the rounded morphology should not be interpreted as an adaptation to prevent collapse of gas-filled spaces, since sucking lice possess very limited internal air volumes and their tracheae are reinforced by taenidia, which mechanically stabilize the tubes against collapse. Recent work further demonstrates that *E. horridus* has a sealed tracheal system and a robust spiracle-closing apparatus, allowing temporary underwater respiration without relying on persistent air stores^71^. Thus, a collapse of large internal air spaces is unlikely to represent a major selective pressure.

The bluish autofluorescent regions revealed in our CLSM data indicate a high proportion of resilin-rich, less sclerotized cuticle in thoracic and abdominal regions in both lice species^60,73^. Anoplura generally possess stretchable cuticle to accommodate substantial blood intake over extended feeding periods^74,75^. In *E. horridus*, these flexible cuticular regions may additionally facilitate slight volumetric changes during submersion while maintaining structural integrity under variable external loads and might represent an adaptation to potential skin respiration under water as an alternative to spiracular breathing on land^54,71,76^ (Fig. 1).

Upon examination of the appendages, it is evident that the legs of the seal louse exhibit a more compact structure compared to that of the terrestrial head louse (Fig. 1C & F). Additionally, in the seal louse, the tibia is fused with the tarsus, forming a tibiotarsus complex^35^. In contrast, the human head louse, along with other terrestrial lice, such as the boar louse (*Haematopinus apri*), displays a distinct separation between the tibia and tarsus^77^. Moreover, the leg of the terrestrial head louse appears to be generally less sclerotized compared to that of the seal louse and the cuticle is also overall thinner. Both species probably exhibit strong sclerotization in their claws; however, in the seal louse, the part of each leg segment closer to the joints is potentially more sclerotized, whereas the segment’s distal portion seems less sclerotized^35^ (Fig. 1). In the head louse, however, these sclerotized regions are not visible, which indicates that the legs might be more flexible overall and probably contain a higher proportion of resilin (Fig. 1C & F)^60,73^. These observations likely reflect the substantially different mechanical environments experienced by the two species. Importantly, *E. horridus* is not embedded deeply within dense insulating fur, as seen in otariids, but occurs predominantly on the head, flippers, and hind flippers of its phocid hosts, where the hair is short and the lice are directly exposed to water flow. In these regions, the boundary layer is very thin during swimming, and local shear and turbulence can exert considerable mechanical stress on attached ectoparasites^35,56–58^. The increased sclerotization in specific parts of the seal louse leg may therefore reinforce the segments that experience the highest external forces, whereas such reinforcement might be unnecessary in the terrestrial head louse to that extent.

The claw shapes also differ significantly between the two Anoplura. While the claw of the head louse is much narrower, more pointed and curved, the claw of the seal louse is much broader, rounder and flatter (Fig. 1C & F). This is probably due to the shape of the individual hairs in seals and in humans. Seal hairs are about 3.5–5.0 times as wide as they are high and are therefore extremely flattened and without large visible surface structures^35^, whereas human head hairs are oval to circular in cross-section and have clear scale structures^78^. In combination with the substantially higher drag forces expected in an aquatic environment and additionally calculated in a previous study using standard hydrodynamic drag equations for seal lice^35^, this might explain the differences in shape and thickness of the claws between the two lice species.

Preuss et al.^35^ had already described the tibial pad and the euplantula in *E. horridus* and their potential contribution to increasing friction on the host hair in detail^35^. The head louse, however, has only one visible pad, the euplantula, whereas a tibial pad is not recognizable. However, the thumb-like counterpart, which has already been described for the seal louse and accommodates the tibial pad, is also much less pronounced in the head louse, suggesting that the associated pad is also reduced or absent (Fig. 1C & F). In other Anoplura, such as the boar louse, no second such pad can be seen^77^. This suggests that this could be a special adaptation of the seal louse to its life in the marine environment and the flat hair of the seals^35^.

### Comparison of leg musculature and locomotion patterns

In the process of reconstructing the anatomical leg structures of the aquatic seal louse and the terrestrial head louse, we identified certain homologies, alongside unexpected deviations from the anticipated fundamental structure of Anoplura, as inferred from the terrestrial head louse. Notably, the claw tendon exhibited a significant difference: in the head louse, it extends through the entire tarsus and tibia to the femur, whereas in the seal louse, it originates at the claw membrane but terminates midway within the tibiotarsus complex (Fig. 3). This phenomenon is highly atypical and, to our knowledge, has not been previously documented. Typically, in other insects, the claw tendon extends at least to the distal end of the tibia, with muscles from the femur attaching to its terminus. This anatomical feature has been documented across a diverse range of insect species, including bugs, beetles, and ants^31,79,80^. In this regard, the terrestrial head louse aligns with the typical anatomical structure of other insects, whereas the seal louse exhibits a significant deviation. The rationale for the seal louse’s shortened claw tendon remains speculative; however, the most plausible explanation is that this adaptation facilitates a more direct and consequently more effective force transmission from the muscles to the claw. A comparable phenomenon has been observed in high-performance athletes, such as sprinters, where a shortened lever arm of the heel generates more force than a longer lever arm in non-sprinters^81^. Furthermore, a shortened lever or tendon reduces the necessity for strong muscle contraction to achieve equivalent tendon and claw movement as with an extended tendon, allowing for lesser energy dissipated in the tendon material during contraction and maintaining the same final power as with a longer tendon. When muscles are required to shorten less and at a slower rate, the body activates a smaller muscle volume to accomplish the same movement, thereby conserving metabolic energy^81–84^. This observation aligns with the finding that seal lice enter a state of akinesis upon contact with water, during which their entire metabolic activity ceases in order to conserve oxygen and energy^54,71,85–87^. Our data do not support the presence of a fully passive self-locking mechanism in the claw of *E. horridus*. Although several structural elements contribute to frictional stabilization once the claw is actively closed, including stopper setae, blade-like ridges on the inner claw surface^35^, and longitudinal ridges on the unguitractor plate (as described for other insects in^31^) (Supplementary Figure S8), these features alone cannot maintain grip in the absence of muscle activity. Among approximately 150 examined individuals, only a small proportion remained attached to hairs after death (own observation), whereas the vast majority detached regardless of preservation method, indicating that claw closure is not sustained once muscular tension is lost.

This interpretation is fully consistent with the functional model described for insect pretarsi, where the *retractor unguis* muscle actively bends the claw via the unguitractor plate, and frictional and elastic elements merely modulate force transmission when external substrate resistance is present^27,30,31,88^. The 3D μCT data presented by^89^ illustrate the internal musculoskeletal arrangement, including the claw flexor muscle, tendon, and unguitractor plate, but do not demonstrate a structural element capable of generating a purely passive locking state. Instead, the claw control system of *E. horridus* matches the established pattern in insects: structural micro-features enhance stability under load, but claw closure requires continuous baseline muscle tension transmitted through the unguitractor plate^31^. The overall architecture therefore reflects an energy-efficient active gripping mechanism rather than a fully passive one, with the shortened claw tendon and the pair of reduced coxal tendons likely helping to minimize the energetic cost of maintaining the baseline tension required for claw closure while still enabling rapid and forceful attachment during active gripping^81^ (Fig. 3).

Overall, the musculature of the seal louse is significantly more pronounced than that of the human head louse (Fig. 3). This is presumably an adaptation to the seal louse’s aquatic lifestyle, as it is exposed to extreme drag forces under water during its host’s dives. This is also reflected in the immense attachment force values measured for the seal louse on seal fur, and this correlate with an exceptionally pronounced musculature^35^. In addition to enhanced musculature, seal lice possess morphological features that can passively reduce drag. Recent work has demonstrated that the dense arrangement of setae on the body surface of *E. horridus* modifies near-surface flow and reduces local drag by disrupting shear and delaying flow reattachment^56^. Although the body of *E. horridus* appears generally rounded, it is not perfectly spherical; in lateral view it is slightly dorsoventrally flattened, a contour that may further contribute to near-surface flow modification without representing a dedicated streamlining adaptation. This mechanism provides a complementary strategy for coping with the hydrodynamic forces acting on the louse during the host’s swimming movements. Thus, the strong leg musculature and claw apparatus likely operate in concert with these passive drag-reducing structures, resulting in a multi-layered mechanical adaptation to the aquatic environment*.*

The general musculature of the two Anoplura species is comparable; however, the seal louse possesses an additional extensor within the tibiotarsus complex, likely facilitating enhanced force transmission to the claw tendon. Furthermore, it exhibits additional protractors and retractors in the femur, which are absent in the head louse (Fig. 3A). Observations of the seal lice locomotion on human head hair and seal hair reveal that the legs demonstrate remarkable flexibility, allowing the lice to maneuver, such that they can ambulate using only three legs on one side of the body (Supplementary Videos S3, S4). Additionally, they can rotate around their own axis on individual human head hair with these three legs (Supplementary Video S4). This suggests a high degree of flexibility within the leg segments, which likely results from the combination of additional protractors and retractors and the resilin-rich, less sclerotized cuticular regions that allow increased joint compliance and enhanced mobility. This flexibility is presumably necessary, in order to be able to switch quickly from one host individual to another, because this is only possible during the haul-outs of the seals on land, and to find a secure grip in the seals’ dense fur^35^. It is striking that they are virtually unable to move on flat rough substrates. In our locomotion experiments, they sat on flat surfaces of varying roughness, groped blindly with their legs, but were unable to grip effectively to the substrate with the tips of their claws (Supplementary Video S2). Thus, their claws seem to represent an extreme adaptation to life on elongated microstructures, such as hairs of their host, whereby loss of contact with the host means certain death, as they can neither swim nor walk on the majority of natural substrates, such as sand or stones^35^. However, they can cling to other materials with elongated geometry, such as human head hair, wires or textile fibers^71^. The only decisive factor for attachment on such fiber-like substrates is that the claws can securely close around a single hair or a bundle of smaller hairs. In the future, it would therefore be interesting to determine the optimal attachment conditions for the seal louse: How do hair diameter, claw closing angle, and the functional roles of the tibial pad and euplantula influence attachment performance?

Beyond these functional aspects, a broader comparative perspective would also be valuable. Examining the remaining echinophthiriid species, parasitizing hosts with differing diving depths and hydrodynamic regimes, could help assess whether the morphological and mechanical patterns identified here represent general aquatic adaptations within the group. Furthermore, comparisons with anopluran species associated with semi-aquatic mammals may provide insight into how intermediate levels of aquatic exposure shape attachment morphology, claw architecture, and leg musculature.

## Conclusions

This study provides the first comprehensive comparative analysis of the leg musculature and attachment mechanisms of the marine seal louse (*E. horridus*) and the terrestrial human head louse (*P. humanus capitis*), focusing on the unique adaptations of the ectoparasitic seal louse to the extreme aquatic environment of its host. Through high-resolution 3D reconstructions and behavioral observations, we demonstrate that *E. horridus* has evolved a suite of morphological and functional specializations, such as a compact body shape, a robust and highly sclerotized tibiotarsus complex, and a shortened claw tendon, that enable secure, reversible attachment to the flattened hair of its diving host under significant hydrostatic pressure and hydrodynamic forces. In contrast to the head louse, the seal louse possesses additional leg muscles (e.g., protractors, retractors, and a second extensor), a fused tibiotarsus, and specialized tendons, indicating an advanced level of functional specialization. These features likely enhance force transmission, reduce energy expenditure during prolonged attachment, and contribute to the exceptionally high attachment forces previously documented. Furthermore, the observed locomotion behavior emphasizes the reliance of *E. horridus* on specific substrate geometries (i.e., hair-like structures) for effective movement and attachment, underscoring its obligate parasitic lifestyle. Altogether, these findings not only deepen our understanding of host-parasite co-adaptations in extreme marine environments, but also provide valuable insights for the development of bioinspired underwater gripping technologies.

## Supplementary Information

Below is the link to the electronic supplementary material.

Supplementary Material 1

Supplementary Material 2

Supplementary Material 3

Supplementary Material 4

Supplementary Material 5

Supplementary Material 6

Supplementary Material 7

Supplementary Material 8

Supplementary Material 9

## Data Availability

All data is provided in the Supplementary Material of the manuscript. Synchrotron data and histological sectioning series^90^ can be provided upon request or online under: [10.6084/m9.figshare.28596953.v2] (https:/doi.org/10.6084/m9.figshare.28596953.v2).

## References

[1] Gorb, S.N. (2001) *Attachment Devices of Insect Cuticle*. 1st edn, *Springer Netherlands*. 1st edn. Dordrecht, Netherlands: Springer, Dordrecht. 10.1007/0-306-47515-4.

[2] Beutel, R. G. & Gorb, S. N. Ultrastructure of attachment specializations of hexapods (Arthropoda): Evolutionary patterns inferred from a revised ordinal phylogeny. *J. Zool. Syst. Evol. Res.* **39**(4), 177–207. 10.1046/j.1439-0469.2001.00155.x (2001).

[3] Gorb, S. N. Biological attachment devices: exploring nature’s diversity for biomimetics. *Philos. Trans. R. Soc. A: Math., Phys. Eng. Sci.* **366**(1870), 1557–1574. 10.1098/rsta.2007.2172 (2008). 10.1098/rsta.2007.2172 18192171

[4] Gorb, S. N. & Beutel, R. Evolution of locomotory attachment pads of hexapods. *Naturwissenschaften* **88**(12), 530–534. 10.1007/s00114-001-0274-y (2001). 11824227 10.1007/s00114-001-0274-y

[5] Bajerlein, D. et al. To attach or not to attach? The effect of carrier surface morphology and topography on attachment of phoretic. *Naturwissenschaften* **103**(7–8), 61. 10.1007/s00114-016-1385-9 (2016). 27379399 10.1007/s00114-016-1385-9 PMC4933732

[6] Federle, W. & Labonte, D. Dynamic biological adhesion: Mechanisms for controlling attachment during locomotion. *Philos. Trans. R. Soc. B: Biol. Sci.* **374**(1784), 20190199. 10.1098/rstb.2019.0199 (2019). 10.1098/rstb.2019.0199 PMC6745483 31495309

[7] Li, D., Huson, M. G. & Graham, L. D. Proteinaceous adhesive secretions from insects, and in particular the egg attachment glue of *Opodiphthera sp.* moths. *Arch. Insect Biochem. Physiol.: Publ. Collab. Entomol. Soc. Am.* **69**(2), 85–105. 10.1002/arch.20267 (2008). 10.1002/arch.20267 18780346

[8] Autumn, K. et al. Adhesive force of a single gecko foot-hair. *Nature* **405**(6787), 681–685. 10.1038/35015073 (2000). 10864324 10.1038/35015073

[9] Autumn, K. et al. Evidence for van der Waals adhesion in gecko setae. *Proc. Natl. Acad. Sci.* **99**(19), 12252–12256. 10.1073/pnas.192252799 (2002). 12198184 10.1073/pnas.192252799 PMC129431

[10] Autumn, K. & Hansen, W. Ultrahydrophobicity indicates a non-adhesive default state in gecko setae. *J. Comp. Physiol. A.* **192**, 1205–1212. 10.1007/s00359-006-0149-y (2006). 10.1007/s00359-006-0149-y 16845535

[11] Autumn, K. & Peattie, A. M. Mechanisms of adhesion in geckos. *Integr. Comp. Biol.* **42**(6), 1081–1090. 10.1093/icb/42.6.1081 (2002). 21680391 10.1093/icb/42.6.1081

[12] Badge, I. et al. The role of surface chemistry in adhesion and wetting of gecko toe pads. *Sci. Rep.* **4**(1), 6643. 10.1038/srep06643 (2014). 25323067 10.1038/srep06643 PMC4200409

[13] Maderson, P. F. A. Keratinized epidermal derivatives as an aid to climbing in gekkonid lizards. *Nature* **203**(4946), 780–781. 10.1038/203780a0 (1964).

[14] Mitchell, C. T. et al. The effect of substrate wettability and modulus on gecko and gecko-inspired synthetic adhesion in variable temperature and humidity. *Sci. Rep.* **10**(1), 19748. 10.1038/s41598-020-76484-6 (2020). 33184356 10.1038/s41598-020-76484-6 PMC7665207

[15] Ruibal, R. & Ernst, V. The structure of the digital setae of lizards. *J. Morphol.* **117**(3), 271–293. 10.1002/jmor.1051170302 (1965). 5883924 10.1002/jmor.1051170302

[16] Williams, E. E. & Peterson, J. A. Convergent and alternative designs in the digital adhesive pads of scincid lizards. *Science* **215**(4539), 1509–1511. 10.1126/science.215.4539.1509 (1982). 17788677 10.1126/science.215.4539.1509

[17] Higham, T. E. & Russell, A. P. Geckos running with dynamic adhesion: Towards integration of ecology, energetics and biomechanics. *J. Exp. Biol.* **228**, 247980. 10.1242/jeb.247980 (2025). 10.1242/jeb.247980 39973192

[18] Russell, A. P. The morphological basis of weight-bearing in the scansors of the tokay gecko (Reptilia: Sauria). *Can. J. Zool.* **64**(4), 948–955. 10.1139/z86-144 (1986).

[19] Chen, Y. et al. Underwater attachment using hairs: The functioning of spatula and sucker setae from male diving beetles. *J. R. Soc. Interface* **11**(97), 20140273. 10.1098/rsif.2014.0273 (2014). 24920108 10.1098/rsif.2014.0273 PMC4208358

[20] Kampowski, T. et al. Exploring the attachment of the Mediterranean medicinal leech (*Hirudo verbana*) to porous substrates. *J. R. Soc. Interface* **17**(168), 20200300. 10.1098/rsif.2020.0300 (2020). 32673516 10.1098/rsif.2020.0300 PMC7423445

[21] Kier, W. M. & Smith, A. M. The structure and adhesive mechanism of octopus suckers. *Integr. Comp. Biol.* **42**(6), 1146–1153. 10.1093/icb/42.6.1146 (2002). 21680399 10.1093/icb/42.6.1146

[22] Smith, A. M. Negative pressure generated by octopus suckers: a study of the tensile strength of water in nature. *J. Exp. Biol.* **157**(1), 257–271. 10.1242/jeb.157.1.257 (1991).

[23] Smith, A. M. Cephalopod sucker design and the physical limits to negative pressure. *J. Exp. Biol.* **199**(4), 949–958. 10.1242/jeb.199.4.949 (1996). 9318745 10.1242/jeb.199.4.949

[24] Busshardt, P. & Gorb, S. N. Walking on smooth and rough ground: Activity and timing of the claw retractor muscle in the beetle *Pachnoda marginata peregrina* (Coleoptera, Scarabaeidae). *J. Exp. Biol.* **216**(2), 319–328. 10.1242/jeb.075614 (2013). 22996445 10.1242/jeb.075614

[25] Dunlop, J. A. Movements of scopulate claw tufts at the tarsus tip of a tarantula spider. *Netherlands J. Zool.* **45**(3–4), 513–520 (1994).

[26] Federle, W. et al. Biomechanics of the movable pretarsal adhesive organ in ants and bees. *Proc. Natl. Acad. Sci.* **98**(11), 6215–6220. 10.1073/pnas.111139298 (2001). 11353847 10.1073/pnas.111139298 PMC33448

[27] Frantsevich, L. & Gorb, S. Structure and mechanics of the tarsal chain in the hornet, *Vespa crabro* (Hymenoptera: Vespidae): implications on the attachment mechanism. *Arthropod Struct. Dev.* **33**(1), 77–89. 10.1016/j.asd.2003.10.003 (2004). 18089024 10.1016/j.asd.2003.10.003

[28] Frazier, S. F. et al. Elasticity and movements of the cockroach tarsus in walking. *J. Comp. Physiol. - A Sensory, Neural, Behav. Physiol.* **185**(2), 157–172. 10.1007/s003590050374 (1999).

[29] Heming, B. S. Functional morphology of the thysanopteran pretarsus. *Can. J. Zool.* **49**(1), 91–108. 10.1139/z71-014 (1971). 5543183 10.1139/z71-014

[30] Niederegger, S. & Gorb, S. N. Tarsal movements in flies during leg attachment and detachment on a smooth substrate. *J. Insect Physiol.* **49**(6), 611–620. 10.1016/S0022-1910(03)00048-9 (2003). 12804721 10.1016/s0022-1910(03)00048-9

[31] Gorb, S. N. et al. The insect unguitractor plate in action: Force transmission and the micro CT visualizations of inner structures. *J. Insect Physiol.* **117**, 103908. 10.1016/j.jinsphys.2019.103908 (2019). 31265818 10.1016/j.jinsphys.2019.103908

[32] Gorb, S. N., Gorb, E. V. & Kastner, V. Scale effects on the attachment pads and friction forces in syrphid flies (Diptera, Syrphidae). *J. Exp. Biol.* **204**(8), 1421–1431. 10.1242/jeb.204.8.1421 (2001). 11273804 10.1242/jeb.204.8.1421

[33] Büscher, T. H. et al. The exceptional attachment ability of the ectoparasitic bee louse *Braula coeca* (Diptera, Braulidae) on the honeybee. *Physiol. Entomol.* **47**(2), 83–95. 10.1111/PHEN.12378 (2022).

[34] Petersen, D. S. et al. Holding tight to feathers – structural specializations and attachment properties of the avian ectoparasite *Crataerina pallida* (Diptera, Hippoboscidae). *J. Exp. Biol.* **221**(13), jeb179242. 10.1242/jeb.179242 (2018). 29712747 10.1242/jeb.179242

[35] Preuss, A. et al. Attachment performance of the ectoparasitic seal louse *Echinophthirius horridus*. *Commun. Biol.* **7**(1), 36. 10.1038/s42003-023-05722-0 (2024). 38182875 10.1038/s42003-023-05722-0 PMC10770372

[36] Bush, A. O. et al. *Parasitism: The diversity and ecology of animal parasites* (Cambridge University Press, Cambridge, 2001).

[37] Kim, K. C. *Coevolution of parasitic arthropods and mammals* (Wiley-Interscience, 1985).

[38] Anderson, R. C. ‘Host-parasite relations and evolution of the Metastrongyloidea (Nematoda)’, *Memoires du Museum National d’Histoire Naturelle*. *Serie A. Zoologie* **123**, 129–132. 10.5281/zenodo.16007555 (1982).

[39] Raga, J. A. et al. Parasites. In *Encyclopedia of Marine Mammals* (eds Perrin, W. F. et al.) 821–830 (Academic Press, London, UK, 2009). 10.1016/B978-0-12-373553-9.00193-0.

[40] Rybczynski, N., Dawson, M. R. & Tedford, R. H. A semi-aquatic Arctic mammalian carnivore from the Miocene epoch and origin of Pinnipedia. *Nature* **458**(7241), 1021–1024. 10.1038/nature07985 (2009). 19396145 10.1038/nature07985

[41] Durden, L. A. & Musser, G. G. The sucking lice (Insecta, Anoplura) of the world - a taxonomic checklist with records of mammalian hosts and geographical distributions. *Bull. Am. Mus. Nat. Hist.* **218**, 1–90 (1994).

[42] Grzimek, B. *Grzimek’s encyclopedia of mammals* (McGraw-Hill Publishing Company, 1990).

[43] Leonardi, M. S. & Palma, R. L. Review of the systematics, biology and ecology of lice from pinnipeds and river otters (Insecta: Phthiraptera: Anoplura: Echinophthiriidae). *Zootaxa* **3630**(3), 445–466. 10.11646/zootaxa.3630.3.3 (2013). 26131525 10.11646/zootaxa.3630.3.3

[44] Eguchi, T. & Harvey, J. ‘Diving behavior of the Pacific harbor seal (*Phoca vitulina richardii*) in Monterey Bay California. *Mar. Mamm. Sci.* **21**, 283–295. 10.1111/j.1748-7692.2005.tb01228.x (2006).

[45] Frost, K. J., Simpkins, M. A. & Lowry, L. F. Diving behavior of subadult and adult harbor seals in Prince William Sound, Alaska. *Mar. Mamm. Sci.* **17**(4), 813–834. 10.1111/j.1748-7692.2001.tb01300.x (2001).

[46] Gjertz, I., Lydersen, C. & Wiig, Ø. Distribution and diving of harbour seals (*Phoca vitulina*) in Svalbard. *Polar Biol.* **24**(3), 209–214. 10.1007/s003000000197 (2001).

[47] Hastings, K. K. et al. Regional differences in diving behavior of harbor seals in the Gulf of Alaska. *Can. J. Zool.* **82**(11), 1755–1773. 10.1139/z04-145 (2004).

[48] Kolb, P. M. A harbor seal Phoca vitulina richardsi, taken from a sablefish trap. *California Fish Game* **68**, 123–124 (1982).

[49] Rosing-Asvid, A. et al. Deep diving harbor seals (*Phoca vitulina*) in South Greenland: movements, diving, haul-out and breeding activities described by telemetry. *Polar Biol.* **43**(4), 359–368. 10.1007/s00300-020-02639-w (2020).

[50] Dehnhardt, G., Mauck, B. & Hyvärinen, H. Ambient temperature does not affect the tactile sensitivity of mystacial vibrissae in harbour seals. *J. Exp. Biol.* **201**(22), 3023–3029. 10.1242/jeb.201.22.3023 (1998). 9787122 10.1242/jeb.201.22.3023

[51] Mauck, B. et al. Thermal windows on the trunk of hauled-out seals: Hot spots for thermoregulatory evaporation?. *J. Exp. Biol.* **206**(10), 1727–1738. 10.1242/jeb.00348 (2003). 12682104 10.1242/jeb.00348

[52] Hansen, S. & Lavigne, D. M. Ontogeny of the thermal limits in the harbor seal (*Phoca vitulina*). *Physiol. Zool.* **70**(1), 85–92. 10.1086/639549 (1997). 9231380 10.1086/639549

[53] Watts, P. Thermal constraints on hauling out by harbor seals (*Phoca vitulina*). *Can. J. Zool.* **70**, 553–560. 10.1139/z92-083 (2011).

[54] Leonardi, M. S. et al. Under pressure: the extraordinary survival of seal lice in the deep sea. *J. Exp. Biol.* **223**(17), jeb226811. 10.1242/jeb.226811 (2020). 32680903 10.1242/jeb.226811

[55] Williams, T. M. & Kooyman, G. L. Swimming performance and hydrodynamic characteristics of harbor seals *Phoca vitulina*. *Physiol. Zool.* **58**(5), 576–589. 10.1086/physzool.58.5.30158584 (1985).

[56] Preuss, A., Gorb, S.N., *et al.* (2025) ‘Role of the Setae in an Ectoparasitic Seal Louse in Reducing Surface Drag: Numerical Modeling Approach’, *Advanced Theory and Simulations*, p. e00429. 10.1002/adts.202500429.

[57] Herzog, I. et al. Heartworm and seal louse: Trends in prevalence, characterisation of impact and transmission pathways in a unique parasite assembly on seals in the North and Baltic Sea. *Int. J. Parasitol.: Parasites Wildlife* **23**, 100898. 10.1016/j.ijppaw.2023.100898 (2024). 10.1016/j.ijppaw.2023.100898 PMC10818207 38283886

[58] Herzog, I., Siebert, U. & Lehnert, K. High prevalence and low intensity of *Echinophthirius horridus* infection in seals revealed by high effort sampling. *Sci. Rep.* **14**(1), 14258. 10.1038/s41598-024-64890-z (2024). 38902289 10.1038/s41598-024-64890-z PMC11190234

[59] Siebert, U. et al. Pathological findings in harbour seals (*Phoca vitulina*): 1996–2005. *J. Comp. Pathol.* **137**(1), 47–58. 10.1016/j.jcpa.2007.04.018 (2007). 17629967 10.1016/j.jcpa.2007.04.018

[60] Michels, J. & Gorb, S. N. Detailed three-dimensional visualization of resilin in the exoskeleton of arthropods using confocal laser scanning microscopy. *J. Microsc.* **245**(1), 1–16. 10.1111/j.1365-2818.2011.03523.x (2012). 22142031 10.1111/j.1365-2818.2011.03523.x

[61] Andersen, S. O. Biochemistry of insect cuticle. *Annu. Rev. Entomol.* **24**(1), 29–59. 10.1146/annurev.en.24.010179.000333 (1979).

[62] Büsse, S. & Gorb, S. N. Material composition of the mouthpart cuticle in a damselfly larva (Insecta: Odonata) and its biomechanical significance. *R. Soc. Open Sci.* **5**(6), 172117. 10.1098/rsos.172117 (2018). 30110404 10.1098/rsos.172117 PMC6030260

[63] Josten, B., Gorb, S. N. & Büsse, S. The mouthparts of the adult dragonfly *Anax imperator* (Insecta: Odonata), functional morphology and feeding kinematics. *J. Morphol.* **283**(9), 1163–1181. 10.1002/jmor.21497 (2022). 35848446 10.1002/jmor.21497

[64] Vincent, J. F. V. Arthropod cuticle: a natural composite shell system. *Compos. A Appl. Sci. Manuf.* **33**(10), 1311–1315. 10.1016/S1359-835X(02)00167-7 (2002).

[65] Cecilia, A. et al. The IMAGE beamline at the KIT light source. *J. Synchrotron Radiat.* **32**(4), 1036–1051. 10.1107/s1600577525003777 (2025). 40455641 10.1107/S1600577525003777 PMC12236244

[66] Douissard, P. A. et al. A versatile indirect detector design for hard X-ray microimaging. *J. Instrum.* **7**(9), P09016. 10.1088/1748-0221/7/09/P09016 (2012).

[67] Vogelgesang, M. et al. Real-time image-content-based beamline control for smart 4D X-ray imaging. *J. Synchrotron Radiat.* **23**(5), 1254–1263. 10.1107/S1600577516010195 (2016). 27577784 10.1107/S1600577516010195

[68] Vogelgesang, M. *et al.* (2012) ‘UFO: A Scalable GPU-based Image Processing Framework for On-line Monitoring’, in *Proceedings of HPCC-ICESS.*, pp. 824–829. 10.1109/HPCC.2012.116.

[69] Faragó, T. et al. Tofu: A fast, versatile and user-friendly image processing toolkit for computed tomography. *J. Synchrotron Radiat.* **29**, 916–927. 10.1107/S160057752200282X (2022). 35511025 10.1107/S160057752200282X PMC9070706

[70] Gray, P. T. A., Mill, P. J. & Dodd, J. M. ‘The musculature of the prothoracic legs and its innervation in *Hierodula membranacea* (Mantidea)’. *Philos. Trans. R. Soc. London B, Biol. Sci.* **309**(1140), 479–503. 10.1098/rstb.1985.0094 (1997).

[71] Preuss, A. et al. The ectoparasitic seal louse, *Echinophthirius horridus*, relies on a sealed tracheal system and spiracle closing apparatus for underwater respiration. *Commun. Biol.* **8**(1), 1–14. 10.1038/s42003-025-08285-4 (2025). 40461816 10.1038/s42003-025-08285-4 PMC12134155

[72] Leonardi, M. S. et al. The deeper the rounder: body shape variation in lice parasitizing diving hosts. *Sci. Rep.* **14**(1), 1–10. 10.1038/s41598-024-71541-w (2024). 39251772 10.1038/s41598-024-71541-w PMC11385217

[73] Appel, E. et al. Ultrastructure of dragonfly wing veins: Composite structure of fibrous material supplemented by resilin. *J. Anat.* **227**(4), 561–582. 10.1111/joa.12362 (2015). 26352411 10.1111/joa.12362 PMC4580113

[74] Vaughan, J. A. & Azad, A. F. Patterns of erythrocyte digestion by bloodsucking insects: constraints on vector competence. *J. Med. Entomol.* **30**(1), 214–216. 10.1093/jmedent/30.1.214 (1993). 8094460 10.1093/jmedent/30.1.214

[75] Waniek, P. J. The digestive system of human lice: Current advances and potential applications. *Physiol. Entomol.* **34**(3), 203–210. 10.1111/j.1365-3032.2009.00681.x (2009).

[76] Leonardi, M. S. et al. Host-parasite coevolution leads to underwater respiratory adaptations in extreme diving insects, seal lice (*Lepidophthirus macrorhini*). *Communications Biology* **8**(1), 1–11. 10.1038/s42003-025-08306-2 (2025). 40467972 10.1038/s42003-025-08306-2 PMC12137713

[77] Soler Cruz, M. D. & Martín Mateo, M. P. Scanning electron microscopy of legs of two species of sucking lice (Anoplura: Phthiraptera). *Micron* **40**(3), 401–408. 10.1016/j.micron.2008.10.001 (2009). 19334295 10.1016/j.micron.2008.10.001

[78] Wolfram, L. J. Human hair: A unique physicochemical composite. *J. Am. Acad. Dermatol.* **48**(6), S106–S114. 10.1067/mjd.2003.276 (2003). 12789162 10.1067/mjd.2003.276

[79] Aibekova, L. et al. The skeletomuscular system of the mesosoma of *Formica rufa* Workers (Hymenoptera: Formicidae). *Insect Systematics and Diversity* **6**(2), 1–26. 10.1093/isd/ixac002 (2022).

[80] Woodworth, C. W. The leg tendons of insects. *Am. Nat.* **42**(499), 452–456. 10.1086/278953 (1908).

[81] Lee, S. S. M. & Piazza, S. J. Built for speed: Musculoskeletal structure and sprinting ability. *J. Exp. Biol.* **212**(22), 3700–3707. 10.1242/jeb.031096 (2009). 19880732 10.1242/jeb.031096

[82] Baxter, J. R. et al. Ankle joint mechanics and foot proportions differ between human sprinters and non-sprinters. *Proc. R. Soc. B: Biol. Sci.* **279**(1735), 2018–2024. 10.1098/rspb.2011.2358 (2012). 10.1098/rspb.2011.2358 PMC3311895 22189400

[83] Fletcher, J. R. & MacIntosh, B. R. Achilles tendon strain energy in distance running: consider the muscle energy cost. *J. Appl. Physiol.* **118**(2), 193–199. 10.1152/japplphysiol.00732.2014 (2015). 25593218 10.1152/japplphysiol.00732.2014 PMC4297774

[84] MacIntosh, B.R. and Holash, R.J. (2000) ‘Power output and force-velocity properties of muscle’, In: B.M. Nigg, B.R. MacIntosh, and J. Mester (Eds) *Biomechanics and biology of movement. Human Kinetics*. Champaign, Illinois (US): Human Kinetics, pp. 193–210.

[85] Kim, K. C. Ecology and morphological adaptations of the sucking lice on the nothern fur seal. *J. Cons. Int. Explor. Mer.* **169**, 504–515 (1975).

[86] Koštál, V. Eco-physiological phases of insect diapause. *J. Insect Physiol.* **52**(2), 113–127. 10.1016/j.jinsphys.2005.09.008 (2006). 16332347 10.1016/j.jinsphys.2005.09.008

[87] Leonardi, M. S. & Lazzari, C. R. Uncovering deep mysteries: The underwater life of an amphibious louse. *J. Insect Physiol.* **71**, 164–169. 10.1016/j.jinsphys.2014.10.016 (2014). 25449903 10.1016/j.jinsphys.2014.10.016

[88] Gorb, S. N. Design of insect unguitractor apparatus. *J. Morphol.* **230**(2), 219–230. 10.1002/(SICI)1097-4687(199611)230:2<219::AID-JMOR8>3.0.CO;2-B (1996). 29852611 10.1002/(SICI)1097-4687(199611)230:2<219::AID-JMOR8>3.0.CO;2-B

[89] Hörger, V., Labisch, S. & Dirks, J.-H. Biomimetic tag attachment inspired by the seal louse. *Bioinspiration Biomimetics* **20**(6), 066015. 10.1088/1748-3190/adfbb8 (2025). 10.1088/1748-3190/adfbb8 40812345

[90] Preuss, A., Schwaha, T., *et al.* (2025a) ‘Nano-CT data and histological sections of *Pediculus humanus capitis* and *Echinophthirius horridus*’, *figshare*. 10.6084/m9.figshare.28596953.v2.

