# Supplementary material for: Functional morphology of the leg musculature in the marine seal louse: adaptations for high-performance attachment to diving hosts
Source: Sci Rep. 2025 Dec 23;15:44732. doi: 10.1038/s41598-025-32804-2 (PMC12749021; doi:10.1038/s41598-025-32804-2)
Supplement: Supplementary file 1 — Supplementary Material 1 [file 41598_2025_32804_MOESM1_ESM.pdf]

Supplementary Material

S1) Supplementary figure of A) a magnified seal louse claw complex and B) a magnified human head louse claw complex both from lateral view. The distinct colors seen in the autofluorescence images are linked to specific material properties (Michels and Gorb, 2012) as follows. Reddish autofluorescence indicates highly sclerotized cuticle, with more intense red hues suggesting greater sclerotization. Greenish autofluorescence is associated with relatively resilient cuticle with a high chitin content, while bluish autofluorescence indicates softer, less-sclerotized regions, often containing resilin. Abbreviations: cl (claw), ep (euplantula), tp (tibial pad).

S2) Supplementary video of a seal louse on a surface of 125 µm roughness filmed from dorsal view.

S3) Supplementary video of a seal louse attaching to seal hair.

S4) Supplementary video of a seal louse attaching with only three legs to a human hair and turning around on the hair.

S5) Supplementary figure of the extrinsic and intrinsic musculature of the first (A) and third leg (B) of *E. horridus.* Musculature of both legs of *E. horridus* with transparent cuticle from lateral view, without cuticle from lateral view and without cuticle from dorsal view. Abbreviations: see Table 1 & Supplementary Table S7; anterior (an), dorsal (d), distal (di), proximal (pr), posterior (ps), ventral (v).

S6) Supplementary figure of the extrinsic and intrinsic musculature of the first (A) and third leg (B) of *P. humanus capitis*. Musculature of both legs of *P. humanus capitis* with transparent cuticle from lateral view, without cuticle from lateral view and without cuticle from dorsal view. Abbreviations: see Table 1 & Supplementary Table S7; anterior (an), dorsal (d), distal (di), proximal (pr), posterior (ps), ventral (v).

S7) Supplementary table for muscle notations used in this manuscript: origin, attachment and function of the extrinsic leg muscles of adult female *E. horridus* and *P. humanus capitis*.

S8) Supplementary figure of the unguitractor plate in *E. horridus*. A) Cross section through the tibiotarsus complex with visible unguitractor plate. B) Dissected unguitractor plate with visible ridges. Abbreviations: cl (claw), ut (unguitractor plate).
