# Supplementary material for: Functional morphology of the leg musculature in the marine seal louse: adaptations for high-performance attachment to diving hosts
Source: Sci Rep. 2025 Dec 23;15:44732. doi: 10.1038/s41598-025-32804-2 (PMC12749021; doi:10.1038/s41598-025-32804-2)
Supplement: Supplementary file 2 — Supplementary Material 2 [file 41598_2025_32804_MOESM2_ESM.pdf]

Supplementary Table S7: Origin, attachment and function of the extrinsic leg muscles of adult female *E. horridus* and *P. humanus capitis*.

Species	Muscle	Origin	Muscle attachment	Function
P. humanus capitis	I/II/IIItbctfl1&2	Posterior, dorsal area of the tarsus	Claw tendon	Flexor of the tarsus
E. horridus	I/II/IIItbtactfl1&2	Posterior, dorsal area of the tibiotarsus	Claw tendon	Flexor of the tibiotarsus
P. humanus capitis	I/II/IIItbcte	Posterior, dorsal area of the tarsus	Claw tendon	Extensor of the claw
E. horridus	I/II/IIItbtacte1&2	Posterior, dorsal area of the tibiotarsus	Claw tendon	Extensor of the claw
P. humanus capitis	I/II/IIIfctfl1&2	Dorsal area of the femur	Claw tendon	Flexor of the tibia
E. horridus	I/II/IIIftbfl1&2	Dorsal area of the femur	Posterior, ventral area of the tibiotarsus	Flexor of the tibiotarsus
P. humanus capitis & *E. horridus*	I/II/IIIftbl	Posterior, dorsal area of the femur	Dorsal area of the tibia/tibiotarsus	Levator of the tibia/tibiotarsus
P. humanus capitis & *E. horridus*	I/II/IIItrfl	Ventral area of the trochanter	Dorsal area of the femur	Levator of the femur
E. horridus	I/II/IIItrfp	Frontal, lateral area of the trochanter	Posterior, lateral area of the femur	Protractor of the femur
E. horridus	I/II/IIItrfr	Abdominal, lateral area of the trochanter	Posterior, lateral area of the femur	Retractor of the femur
P. humanus capitis	I/II/IIIcctl1&2	Ventral area of the coxa	Coxal tendon	Levator of the trochanter
E. horridus	I/II/IIIcdctl1&2	Ventral area of the coxa	Dorsal coxal tendon	Levator of the trochanter
P. humanus capitis	I/II/IIIcctd1&2	Ventral area of the coxa	Coxal tendon	Depressor of the trochanter
E. horridus	I/II/IIIcvctd1&2	Ventral area of the coxa	Ventral coxal tendon	Depressor of the trochanter
P. humanus capitis & *E. horridus*	I/II/IIIfucl	Central body furca	Dorsal area of the coxa	Levator of the coxa
P. humanus capitis & *E. horridus*	I/II/IIIfucfl	Central body furca	Coxal tendon	Flexor of the coxa
P. humanus capitis & *E. horridus*	I/II/IIIfucp	Central body furca	Frontal lateral area of the coxa	Protractor of the coxa
P. humanus capitis & *E. horridus*	I/II/IIIfucr	Central body furca	Abdominal lateral area of the coxa	Retractor of the coxa
