# Supplementary figures and images for: Functional morphology of the leg musculature in the marine seal louse: adaptations for high-performance attachment to diving hosts
Source: Sci Rep. 2025 Dec 23;15:44732. doi: 10.1038/s41598-025-32804-2 (PMC12749021; doi:10.1038/s41598-025-32804-2)

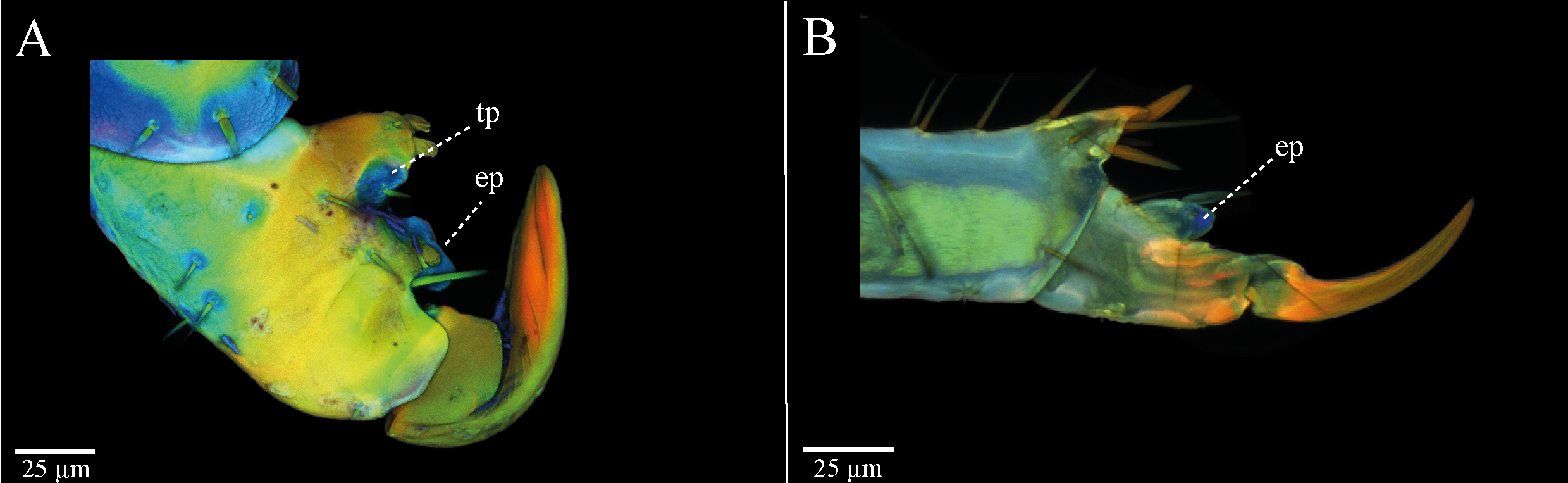

Supplement: Supplementary file 3 — Supplementary Material 3 [file 41598_2025_32804_MOESM3_ESM.tif]

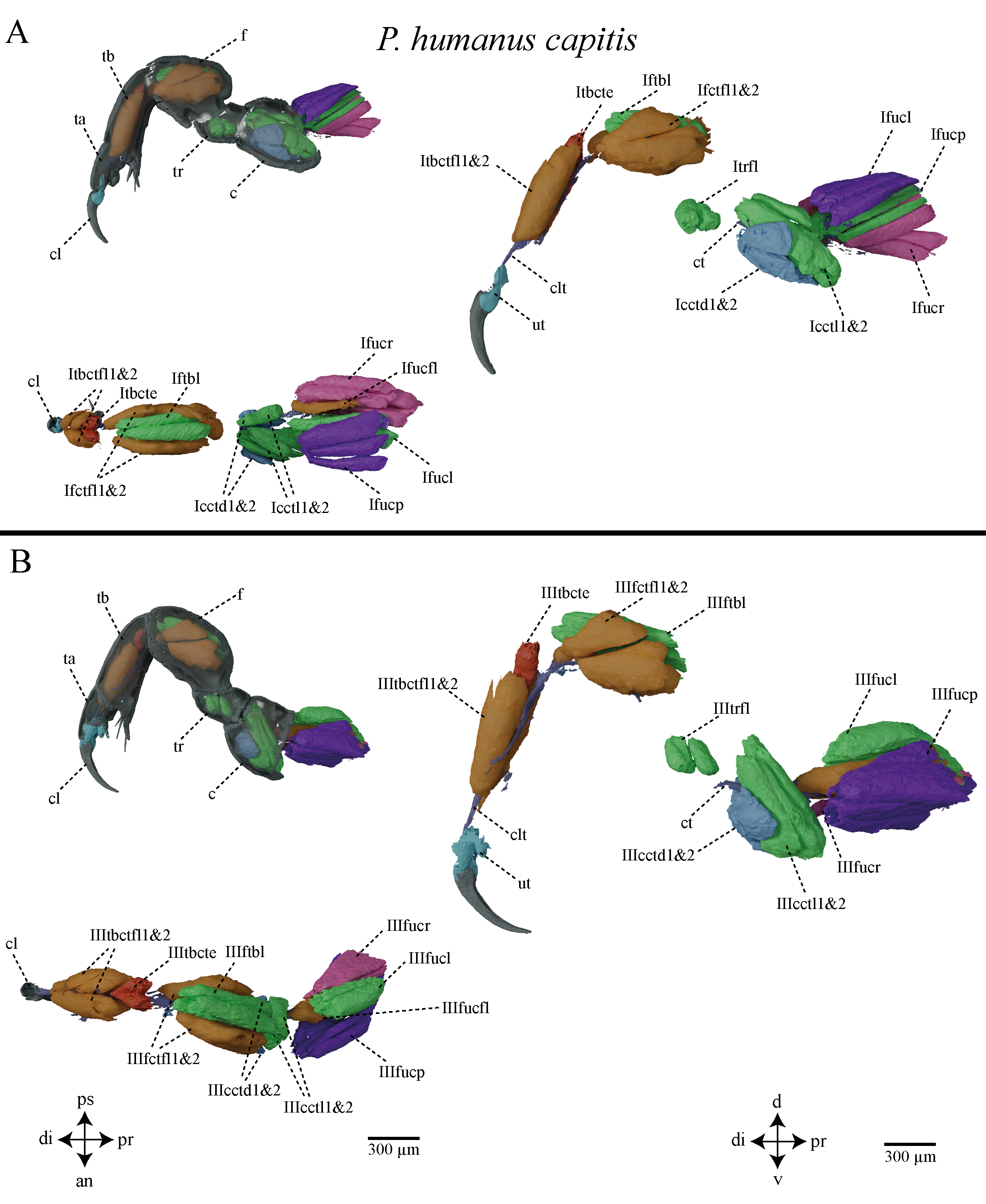

Supplement: Supplementary file 7 — Supplementary Material 7 [file 41598_2025_32804_MOESM7_ESM.tif]

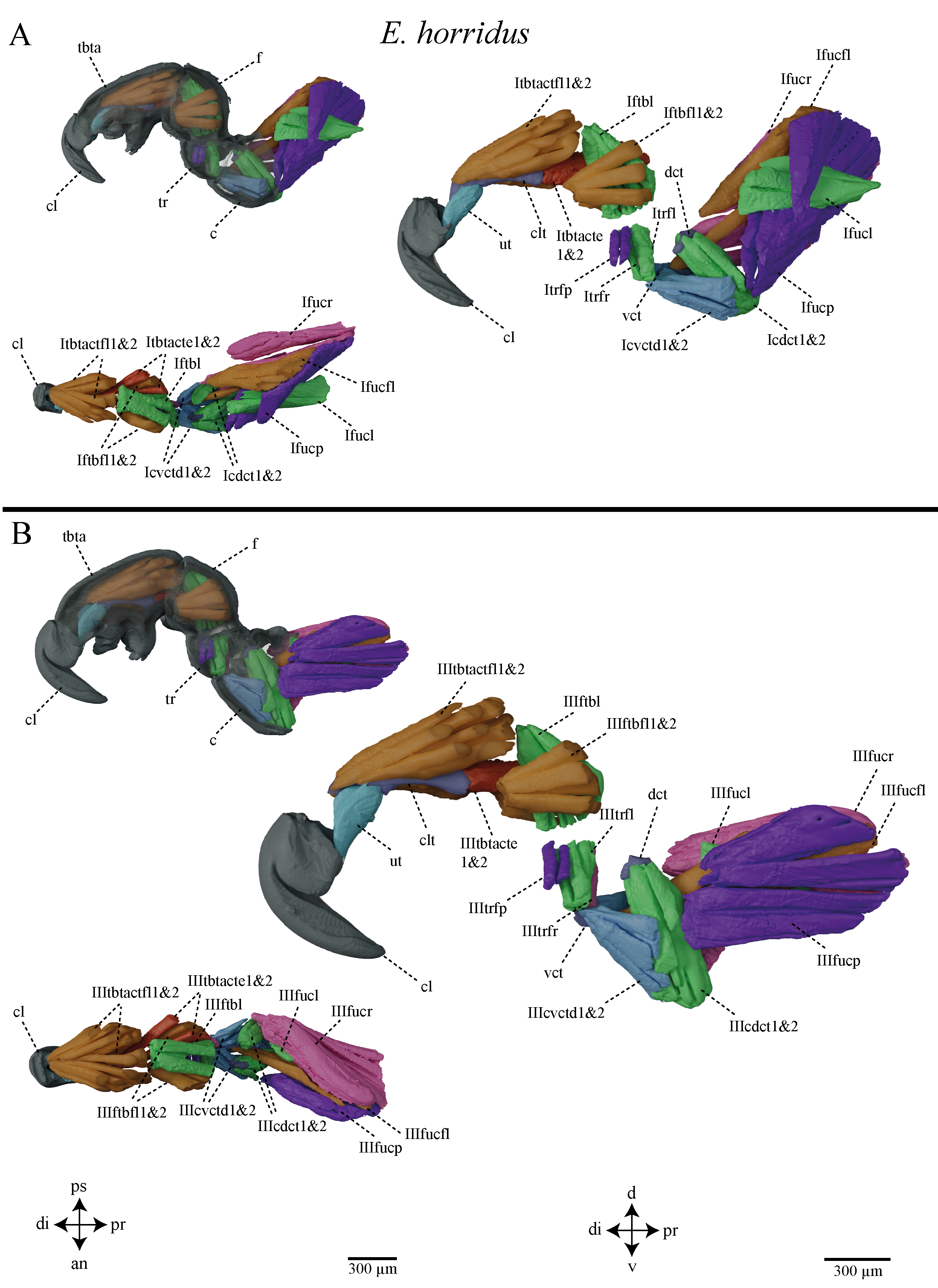

Supplement: Supplementary file 8 — Supplementary Material 8 [file 41598_2025_32804_MOESM8_ESM.tif]

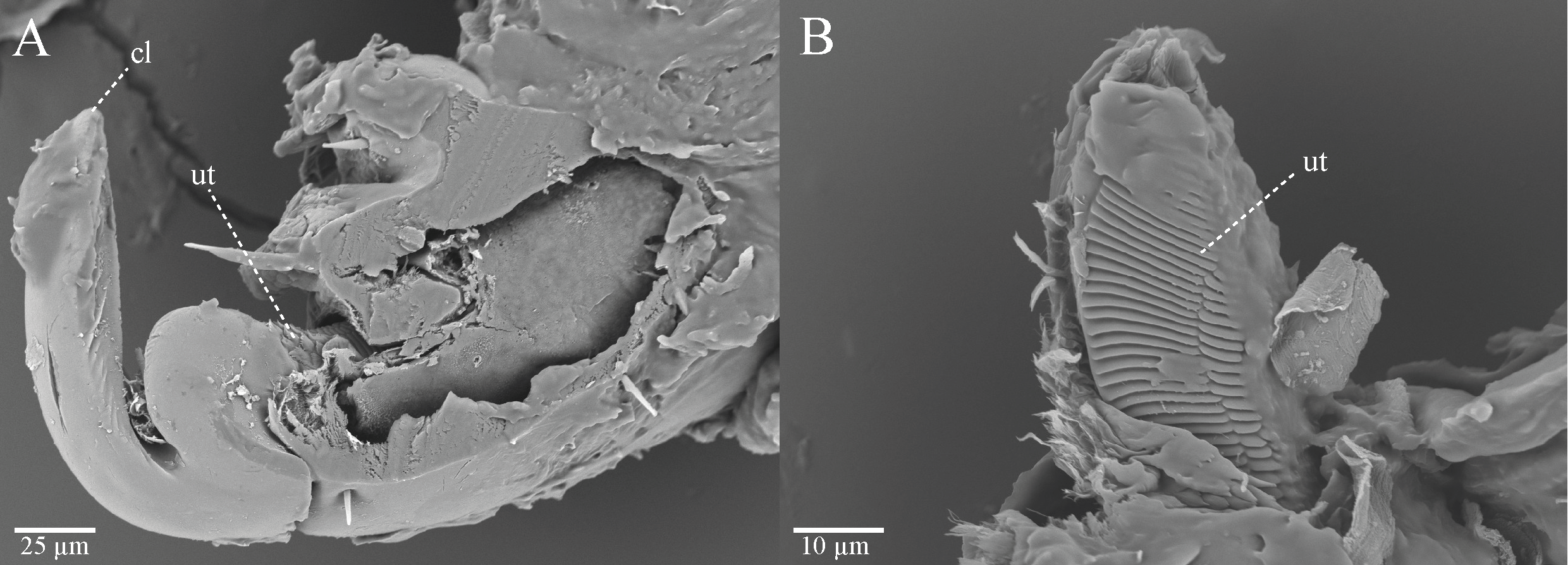

Supplement: Supplementary file 9 — Supplementary Material 9 [file 41598_2025_32804_MOESM9_ESM.tif]
